# The ED50 and ED95 of esketamine for preventing early postoperative pain in patients undergoing laparoscopic cholecystectomy: a prospective, double-blinded trial

**DOI:** 10.1186/s12871-023-02357-w

**Published:** 2023-11-25

**Authors:** Zhongling Xu, Yantao Lang, Xiaolei Xu, Linjuan Deng, Hengya Song, Dekun Yin

**Affiliations:** 1 Department of Anesthesiology, Affiliated Hospital of Nantong University, Nantong, 226001 Jiangsu province China; 2 Department of Anesthesiology, Funing People’s Hospital of Jiangsu, Yancheng, 224400 Jiangsu province China

**Keywords:** Esketamine, ED50, ED95, Postoperative pain, Laparoscopic cholecystectomy

## Abstract

**Background:**

This study aims to estimate the safety, efficacy, and median effective dose (ED50) of esketamine for preventing early postoperative pain in patients undergoing laparoscopic cholecystectomy.

**Methods:**

54 patients undergoing laparoscopic cholecystectomy were prospectively randomized into two groups (group C and group E). Different doses of esketamine were intravenously administered before the skin incision in Group E. The patients in group C received the same dose of saline at the same time. General population characteristics were recorded. The median effective dose (ED50) and 95% effective dose (ED95) were calculated using Dixon’s up-and-down method. Hemodynamic parameters were monitored, and pain intensity was assessed using a visual analog scale. We also recorded the condition of anesthesia recovery period and postoperative adverse reactions.

**Results:**

The ED50 of esketamine for preventing early postoperative pain was 0.301 mg/kg (95%CI: 0.265-0.342 mg/kg), and the ED95 was 0.379 mg/kg (95%CI: 0.340-0.618 mg/kg), calculated by probability unit regression. Heart rate (HR) was significantly lower in the esketamine group compared to the control at the skin incision (*p < 0.05*). The total VAS score at resting was significantly lower in the esketamine group compared to the control group during the awakening period (*p < 0.05*). There was no significant difference between the two groups regarding the incidence of adverse reactions (*p > 0.05*).

**Conclusions:**

In this study, esketamine can prevent early postoperative pain effectively. The ED50 and ED95 of esketamine for controlling early postoperative pain were 0.301 mg/kg and 0.379 mg/kg, respectively.

**Trial registration:**

ChiCTR2200066663, 13/12/2022.

## Introduction

Laparoscopic cholecystectomy (LC) has been the gold standard for surgical treatment of gallbladder disease because of short surgery time, good surgical vision, less trauma, less blood loss, easy recovery, and low infection rate [1, 2]. However, acute early postoperative pain after LC is a kind of acute nociceptive response, which usually causes severe physical discomfort in patients accompanied by hemodynamic and metabolic instability, prolonging recovery and delaying discharge, mainly comprising incisional pain, visceral pain, and opioid-induced hyperalgesia(OIH) [3–5]. Incisional pain, one of the essential components of acute early postoperative pain, is defined as superficial pain on the abdominal wall, which can be blocked by neuraxial block, peripheral nerve block, and local infiltration [3]. Different from incisional pain, visceral pain is defined as pain inside the abdomen, which may be deep, dull, and more difficult to localize. The visceral pain after LC surgery is mainly caused by surgical handling, diaphragmatic irritation from dissolved carbon dioxide, peritoneal inflammation, local acidosis, and visceral mucosa ischemia [5–7]. On the one hand, Morten et al. [8] concluded that high VAS scores for early visceral pain were associated with chronic unexplained pain in patients undergoing LC. Chronic visceral pain also induces peripheral and central sensitization more frequently than incisional pain [9, 10]. On the other hand, visceral pain is accompanied by symptoms arising from the autonomic and enteric nervous system [9, 11], including nausea and gastrointestinal disturbances, which may be aggravated by general anesthetics-induced postoperative nausea and vomiting (PONV). Therefore, finding an effective method to relieve visceral pain is particularly important for controlling acute early postoperative pain in LC patients. Meanwhile, hyperalgesia is another essential part of postoperative pain that manifests with a lower threshold for mechanical/pressure/cold stimulation following remifentanil use, which is a state of nociceptive sensitization (excessive pain from mildly noxious stimuli or pain caused by non-noxious stimuli) [19, 20]. Several experimental and clinical studies suggested that despite opioids being associated with varying degrees of hyperalgesia, short-acting opioids (such as remifentanil) seemed to induce OIH more rapidly and frequently than longer-acting opioids [12–14]; and allodynia area unassociated with the injury site was significantly enlarged after remifentanil intravenous infusion [15, 16]. The presence of hyperalgesia in the acute postoperative period is likely to increase the amount of pain experienced [12]. Therefore [25], the prevention of opioid-induced hyperalgesia is of great importance for acute early postoperative pain after LC.

Ketamine, a noncompetitive, channel-blocking NMDA-R antagonist, is considered to be highly correlated with acute early postoperative pain (incisional pain, visceral pain, and OIH). The studies indicated that preemptive ketamine might reduce acute early postoperative pain, especially incisional pain and visceral pain, in patients undergoing gynecological laparoscopic surgery [17, 18]. In parallel, the ketamine (5 μg/kg per minute) was confirmed to reduce remifentanil-induced hyperalgesia (RIH) at a site distant from the primary injury but not at the incision site [16, 12, 13, 23, 26, 28]. As is well known, ketamine (or RS-ketamine) is a racemic mixture containing equal parts of R-ketamine and S-ketamine (esketamine). Therefore, theoretically, esketamine can also have a direct inhibitory role on acute early postoperative pain (incisional pain, visceral pain, and OIH). Some of the recent research showed that intraoperative administration of esketamine reduced perioperative sufentanil consumption, acute and chronic postoperative pain, and postoperative rebound pain, which could improve the quality of recovery [19–21]. However, high-dose esketamine induces several adverse effects, such as circulatory, psychiatric, and neurological systems. For instance, Min Zhu et al. [21] found that the high-dose esketamine (0.5 mg/kg loading, 4 μg/kg/h infusion) increased incidence of drowsiness and postoperative bispectral index value (psychotomimetic-related brain electrical activities) significantly. In striking contrast, esketamine (0.5 mg/kg loading, 0.24 mg/kg/h infusion) did not increase psychotomimetic side effects in patients undergoing thyroidectomy in Penglei Wang et al. [22]. Based on the aboved-mentioned, there are some potentially significant clinical implications to determining the optimal dose of esketamine for preventing early postoperative pain and controlling esketamine-induced adverse effects. Furthermore, previous literature reported that the ED50 of esketamine was 0.143 mg/kg when combined with 3 mg/kg propofol for successful sedation by the up-down sequential method [23], a simple and classical method in the dose-effect relationship research. Therefore, we plan to use the Dixon up and down method to determine ED50 of esketamine for providing adequate analgesia with the lowest number of adverse drug reactions.

In this study, we estimate the safety and efficacy of esketamine for preventing postoperative pain in patients undergoing laparoscopic cholecystectomy, and determine the median effective dose(ED50) and 95% effective dose (ED95) of esketamine by applying the up-and-down sequential allocation technology designed by Dixon and Massey.

## Methods

### Design and patients

We performed a prospective, double-blind, up-down sequential allocation study to determine the ED50 of esketamine for preventing early postoperative pain. The study was conducted in agreement with the Helsinki Declaration and approved by the Ethics Committee of Affiliated Hospital of Nantong University. All participants underwent an informed consent process and signed a consent form. The study was registered in the Chinese Trial Registry (ChiCTR2200066663, 13/12/2022). This study adhered to the CONSORT 2010 statement.

The patients who were scheduled to receive laparoscopic cholecystectomy were enrolled in the study. The inclusion criteria for the studies were as follows: (1) age: ≥18 years old, (2) BMI: 18–28 kg/m^2^, (3) no restrictions on gender and ethnicity, (4) ASA class I and II, (5) all patients must have total comprehension. Exclusion criteria were as follows: (1) immediate extubation was not planned after surgery, (2) patients were complicated with substantial diseases of essential organs, (3) allergy to related drugs applied in the trial, (4) patients with a history of alcohol or drug abuse, (5) the patients had severe hypokalemia, ocular hypertension and intracranial hypertension, (6) participants had a history of psychiatric neurological disorders and/or taking the related medicine, (7) intraoperative changeover from laparoscopic to open cholecystectomy.

### Randomization, blinding and allocation concealment

66 patients requiring general anesthesia were randomized into the control group (group C) or esketamine group (group E) using a computer-generated random sequence placed in sequentially numbered opaque envelopes, which were opened before surgery. The drugs were prepared in 20-ml syringes labeled as the “study drug” and then placed into opaque envelopes before the study by an independent anesthesiologist. Another experienced anesthesiologist implemented anesthesia, and a specialized anesthesia nurse, blinded to the group allocation, performed the postoperative data collection. Patients, clinicians, and nurses were blinded to group assignment and study drugs for the duration of the trial. Incisional pain is defined as a pain restricted to incision site on abdominal wall. Visceral pain is defined as a deep, dull and hard-to-locate pain, while opioid-induced hyperalgesia (OIH) is defined as an increased sensitivity to normally painful stimuli in LC. Patients were educated to distinguish these three types of pain from our definitions.

### Anesthetic management

The anesthesia and surgical procedure will be consistent between the two groups. In the operating room, the patient received an intravenous fluid replacement and mask oxygen. Then, vital signs and depth of anesthesia were monitored, and baseline values were measured before anesthesia. General anesthesia was induced with midazolam 0.03-0.04 mg/kg, etomidate 0.2–0.3 mg/kg, sufentanil 0.3–0.5 μg/kg, and cisatracurium 0.15-0.2 mg/kg. After tracheal intubation, Group E received different doses of esketamine through intravenous administration, and Group C received the same quantity of saline at the same time. The anesthesia was maintained with propofol 4–6 mg/(kg·h), sevoflurane 1-1.5%, remifentanil 0.2–0.3 μg/(kg·min), and cisatracurium 0.1-0.15 mg/kg/h. Sevoflurane and cisatracurium were no longer used during skin incision suturing. Inadequate anesthesia was defined as patient movement, swallowing, lacrimation, sweating, hypertension, and tachycardia (more than 20% above baseline values for at least 1 min), and the bispectral index (BIS) score greater than 60. When the depth of anesthesia was considered insufficient, the propofol, remifentanil and sevoflurane infusion/intake rates were changed in steps of 1 mg/(kg·h), 0.05 μg/(kg·min) and 0.5%, respectively. BP and HR were stabilized between ± 20% of baseline. Parecoxib 40 mg was administered when pneumoperitoneum was stopped, and 0.5% ropivacaine was applied to the incision for local infiltration anesthesia.

Atropine and esmolol were routinely administered to regulate Heart rate (HR), and dopamine and urapidil were used for maintaining blood pressure stability conventionally. All anesthetics were discontinued after the operation, and then the patients were transferred to the postanesthetic care unit (PACU). If the TOF ratios were 1.0, residual neuromuscular blockade was antagonized with neostigmine 0.02 mg/kg together with atropine 0.01 mg/kg, and the dosages of drugs were adjusted based on the patient’s actual conditions. The patients were extubated after surgery when they met the extubation indications.

### Study intervention and determination of the ED50 of esketamine

Group E received different doses of esketamine (Jiangsu Hengrui Medicine Co., Ltd.) through intravenous administration before the skin incision. The patients in group C received the same quantity of saline at the same time. The EC50 and ED95 of esketamine for the patient were determined by the up-down allocation methodology [24].

**Fig. 1:**
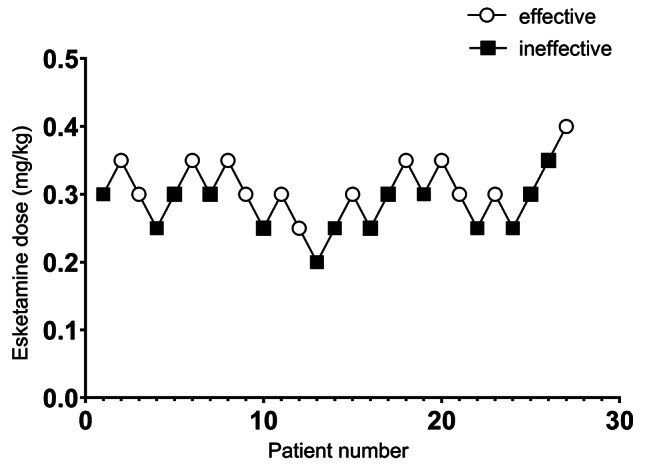
Dixon and Massey up-and-down plot line chart. The sequence of patients receiving esketamine in Group E. The quality of analgesia was measured using VAS (from 0 to 10) and was defined as ineffective (VAS score ≥ 3) or effective (VAS score<3)

The starting dose of esketamine was set at 0.3 mg/kg for the first participant, as determined by drug instructions. This dose is commonly used in clinical practice and many studies [25, 26]. The dose adjustment space was 0.05 mg/kg. All the patients were asked to score their pain on a visual analog scale (VAS) score at 15 min after tracheal extubation. Pain assessment was blind. When the VAS score at rest was less than 3, the analgesia was considered effective. When the VAS score at rest was greater than or equal to 3, the analgesia was considered ineffective. The dose for the following patient was decided by the analgesic effect of the previous one. If a patient’s response was ineffective, the esketamine dose given to the next patient was increased by 0.05 mg/kg. If the response was effective, the esketamine dose given to the next patient was decreased by the same amount. According to Dixon’s up-down method, new patients will no longer be included in the study when 6–8 crossing points (effective turned to ineffective) appear in Fig. 1. In postoperative analgesia with VAS score greater than 4, morphine 0.1 mg/kg was administered for rescue analgesia.

### Type of outcome measures

The primary outcome measure was the ED50 and ED95 of esketamine for preventing early postoperative pain. The visual analogue scale (VAS) was used to measure the pain intensity at the time point of the awakening period (15 min after tracheal extubation) in two groups, which was an 11 cm scale from zero (no pain) to 10 (most severe pain).

The secondary outcome measures were demographic data, vital signs, the condition of patients during anesthesia recovery, and postoperative adverse reactions. Demographic data were recorded, including age, gender, body mass index(BMI), ASA classification (I/II), duration of surgery, sufentanil doses, remifentanil doses, and propofol doses(intraoperative maintenance doses). Vital signs (the mean arterial pressure, MAP; heart rate, HR) were tabulated at the time point of T0 (pre-anesthesia, baseline), T1 (skin incision), and T2 (the dissection of the gallbladder off the liver bed by electric knife). There were also several indexes during the anesthesia recovery: awakening time (the time interval from the end of surgery to extubation), VAS score(incisional pain, resting and cough) at 15 min after tracheal extubation, total VAS score(resting and cough) at 15 min after tracheal extubation, Steward score after extubation, numbers requiring rescue analgesia after extubation, numbers requiring antagonize the residual muscle relaxant, and the medication usage conditions (especially narcotics) in PACU. The postoperative adverse reactions included nausea and vomiting (PONV), hallucinosis, dizziness, itching, nystagmus, nightmare, and postoperative respiratory depression.

Steward Scale score is usually applied to assess the awakening intensity during the recovery period and mainly includes awake degree, airway patency, and limb mobility. The awake degree is divided into three categories: fully awake (2 points), response to stimulation (1 point), and no response to stimulation (0 point). Airway patency is collapsed into three categories: can cough according to the doctor’s order (2 points), can maintain airway patency without support (1 point), and respiratory tract needs support (0 point). Limb mobility is classified into three categories: limbs can do conscious activities (2 points), limbs can make unconscious activities (1 point), and no movement of limbs (0 point). The patients could be thought to be leaving the recovery room when the overall scores are greater than or equal to 4.

### Statistical analyses

The data were processed by the software SPSS 22.0 and graphed by GraphPad Prism 9.5.1. Normally distributed continuous data were presented as mean ± standard deviation (SD) and analyzed using the Student’s t-test. Non-normally distributed continuous data were expressed as median (IQR) and analyzed using the Mann-Whitney U test. Categorical variables were expressed as frequencies (percentages) and analyzed using the Chi-square test (χ^2^) or Fisher’s exact test. The ED50, ED95, and 95% confidence intervals (CIs) of esketamine were calculated using the probit method (probability unit regression) by analyzing the tallied numbers of ‘effective’ and ‘ineffective’ responses for each group based on the up-and-down allocation method.

## Results

**Fig. 2:**
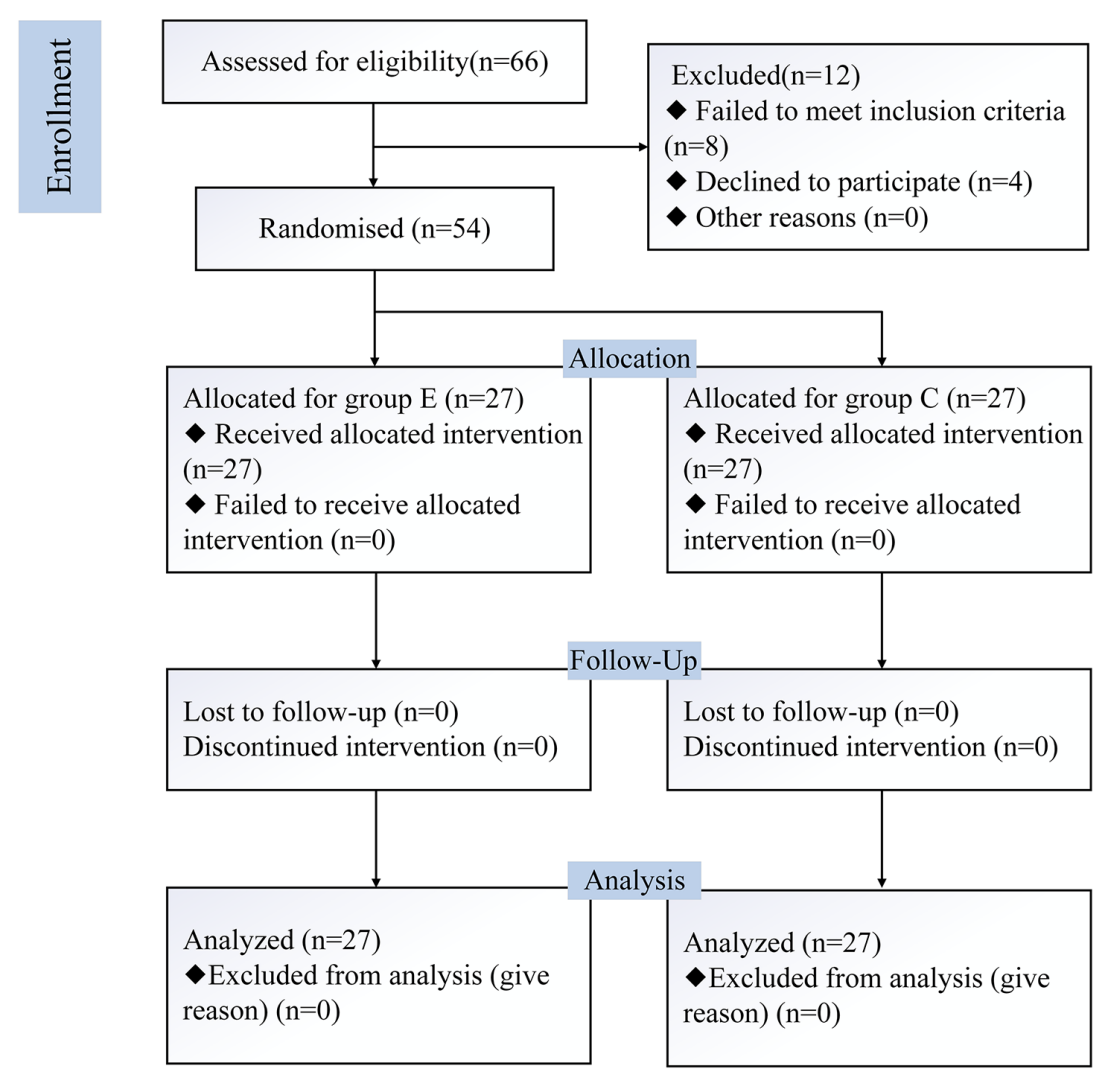
Flow diagram

Of the 66 laparoscopic cholecystectomy patients who were screened for inclusion, 8 met exclusion criteria, 4 refused to participate, and 54 met the inclusion criteria and were randomized into two groups of 27 patients each (Fig. 2). There were no statistically significant differences between the two groups for the demographic characteristics, including age, gender, body mass index(BMI), ASA classification, duration of surgery, sufentanil doses, remifentanil and intraoperative propofol doses (*p > 0.05*) (see Table 1).

**Table 1:** Demographic data and intraoperative outcomes

Index	Group E(n = 27)	Group C(n = 27)	χ^2^/t/z value	*P* value
Age (year)	56(41, 67)	55(51, 61)	-0.009	0.993
Gender (male/female)	8/19	9/18	0.086	0.770
BMI (kg/m^2^)	23.74(23.15, 24.46)	22.86(22.10, 24.57)	-1.479	0.139
ASA classification (I/II)	11/16	13/14	0.300	0.584
Duration of surgery (min)	41(35, 53)	40(36, 50)	-0.026	0.979
Sufentanil doses (μg)	28.44 ± 4.65	27.15 ± 4.35	1.058	0.833
Remifentanil doses (μg)	608(488, 796.5)	631(490, 764.5)	-0.156	0.876
Propofol doses (mg)	196.5(148.5, 251)	205(161, 263)	-0.493	0.622

Notes: ASA, American Society of Anesthesiologists; BMI, body mass index; Propofol doses, intraoperative maintenance doses. Data are expressed as number, the mean ± standard deviation or medians (quartiles). Measurement data is treated with the t-test, or Mann-Whithey U test, enumeration data is treated with the chi-square test

**Fig. 3:**
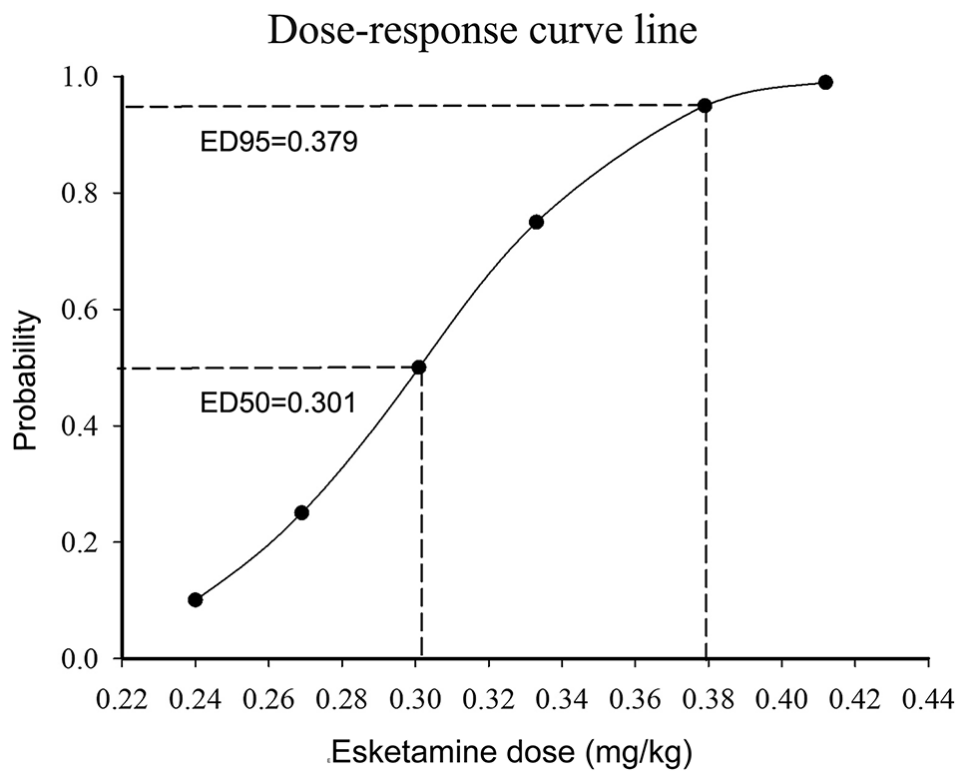
Dose-response curve of esketamine for preventing early postoperative pain in patients undergoing laparoscopic cholecystectomy

The sequence of effective and ineffective determined by Dixon’s up-and-down method is shown in Fig. 1, and the ED50 values are presented in Fig. 3. Using the probability unit regression, we found that the ED50 of esketamine for preventing early postoperative pain was 0.301 mg/kg (95%CI: 0.265-0.342 mg/kg), and the ED95 was 0.379 mg/kg (95%CI: 0.340-0.618 mg/kg).

**Fig. 4:**
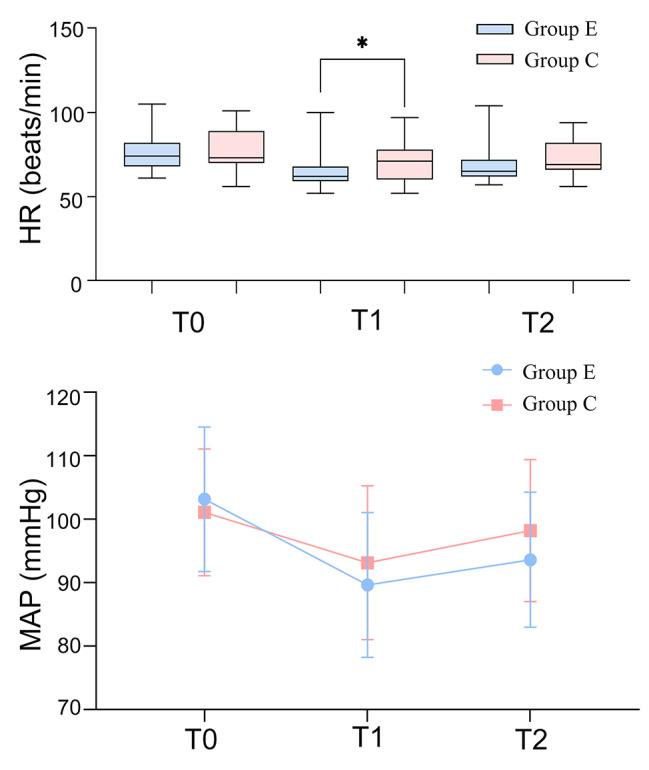
Heart rate (HR) and mean arterial pressure (MAP) at different time points during surgery. Data are expressed as mean (standard deviation) or median (interquartile range). T_0_: BL, baseline; pre-operation. T_1_: at the time of skin incision. T_2_: the gallbladder dissection off the liver bed by the electric knife. * *p* < 0.05 vs. control group

The mean arterial pressure (MAP) and heart rate (HR) are shown in Table 2; Fig. 4. There were no statistically significant differences in MAP between the two groups at T0, T1, and T2(*p > 0.05*). No significant differences in HR values were found between the two groups at the time points of T0 and T2(*p > 0.05*). Notably, there was a significant increase in heart rate in the control group at the time points of T1 compared to the esketamine group (*p < 0.05*).

**Table 2:** The intraoperative haemodynamics condition

Index	Group E(n = 27)	Group C(n = 27)	t/z value	*P* value
HR (beats/min)				
T0	74(68, 82)	73(70, 89)	-0.745	0.456
T1	62(59, 68) *	71(60, 78)	-1.985	0.047
T2	65(62, 72)	69(66, 82)	-1.767	0.077
MAP (mmHg)				
T0	103.17 ± 11.39	101.11 ± 9.99	0.707	0.643
T1	89.63 ± 11.42	93.14 ± 12.13	-1.094	0.900
T2	93.62 ± 10.65	98.22 ± 11.21	-1.547	0.956

Notes: Data are expressed as mean (standard deviation) or median (interquartile range). T_0_: BL, baseline; pre-operation. T_1_: skin incision. T_2_: the dissection of the gallbladder off the liver bed by electric knife. * *p* < 0.05 vs. control group

The conditions of patients during anesthesia recovery are listed in Table 3. There were no statistically significant differences between the two groups in awakening time, incisional pain-related VAS score at resting, incisional pain-related VAS score during cough, total VAS score during cough, Ramsay score, the numbers requiring rescue analgesia, numbers requiring to antagonize the residual muscle relaxant, and the doses of narcotics (including morphine, neostigmine, and atropine) during the awakening period (*p > 0.05*). Compared with the control group, there was a significant decrease in the total VAS score at resting during the awakening period in the esketamine group (*p < 0.05*).

**Table 3:** The condition of patients during anesthesia recovery

Index	Group E(n = 27)	Group C(n = 27)	χ^2^/z/t value	*P* value
Awakening time (min)	22(20, 24)	23(21, 25)	-1.158	0.247
VAS score(incisional pain)				
Resting	0(0, 0)	0(0, 0)	0	1
Movement (cough)	0(0, 0)	0(0, 0)	0	1
total VAS score				
Resting	3(2, 3) *	3(3, 4)	-2.208	0.027
Movement (cough)	4(2, 4)	4(3, 5)	-1.405	0.160
Steward score	5(4, 5)	5(4, 5)	-0.418	0.676
Numbers requiring rescue analgesia, n (%)	6 (22.22%)	10 (37.04%)	1.421	0.233
Morphine (mg)	6.30 ± 1.24	6.32 ± 0.80	-0.035	0.221
Numbers requiring antagonize the residual muscle relaxant, n (%)	10 (37.04%)	6 (22.22%)	1.421	0.233
Neostigmine(mg)	1.00 ± 0.24	0.83 ± 0.26	1.291	0.202
Atropine(mg)	0.53 ± 0.18	0.42 ± 0.13	1.378	0.809

Notes: VAS, Visual Analogue Scale/Score. Data are expressed as number, the mean ± standard deviation or medians (quartiles). Measurement data is treated with the t-test, or Mann-Whithey U test, enumeration data is treated with the chi-square test. * *p < 0.05* vs. control group

Postoperative adverse events as one of the outcomes are listed in Table 4. We found no patient presented with hallucinosis, itching, nystagmus, nightmares, or postoperative respiratory depression in either group. No statistically significant differences between the two groups were found with regard to PONV and dizziness (*p > 0.05*).

**Table 4:** The condition of post-operative adverse reactions

Index	Group E(n = 27)	Group C(n = 27)	χ^2^ value	*P* value
PONV, n (%)	3 (11.11%)	6 (22.22%)	1.200	0.273
Hallucinosis, n (%)	0	0	/	/
Dizziness, n (%)	2 (7.41%)	3 (11.11%)	0.220	0.639
Itching, n (%)	0	0	/	/
Nystagmus, n (%)	0	0	/	/
Nightmare, n (%)	0	0	/	/
Postoperative respiratory depression, n (%)	0	0	/	/

Notes: PONV, Postoperative Nausea and Vomiting. Data are expressed as number (percentage). Enumeration data is treated with the chi-square test

## Discussion

Biliary calculus, also known as gallstone, is a crystalline solid formed from bile concentration and composition changes caused by changes in diet, hormones, medications, or rapid weight loss or weight gain [27]. Laparoscopic cholecystectomy is the standard treatment for symptomatic cholelithiasis, especially when it is complicated by sharp, constant abdominal pain, fever, nausea, and vomiting [1, 2, 27]. Severe acute postoperative pain following LC is still prevalent due to the particularly complex nerve patterns distributed, electrocautery-associated injuries, incision injuries, and opioid-induced hyperalgesia [3–5]. Currently, the medications, preventing peri-and postoperative pain, mainly include opioid and non-opioid analgesics. Despite irrefutable clinical application in pain management, opioid use is sometimes restricted by many undesirable adverse effects, such as opioid dependence, tolerance, constipation, itch, or respiratory depression [28]. Opioid-free anesthesia modalities, represented by ketamine and ketamine, have become an attractive alternative for peri-and postoperative analgesia.

Esketamine, the S (+)-isomer of ketamine, a non-selective NMDA receptor inhibitor, possesses part of non-opioid analgesic properties and an analgesic effect twice that of racemic ketamine [29, 30], which is a dominant agent in opioid-free anesthesia for a long time [31]. Although esketamine has been demonstrated to be effective in controlling post-laparoscopy pain, many associated negative side effects may correlate with its dose intensity [21, 22, 31]. Therefore, we determine the ED50 of esketamine to explore an equilibrium point between the most excellent clinical effect and the lowest adverse reactions. For the starting dose selection, different studies use different dose settings, mainly including 0.1 mg/kg, 0.2 mg/kg, 0.3 mg/kg, and several other doses. For instance, the first child was given an esketamine dose of 0.1 mg/kg in order to explore the ED50 of esketamine for children to inhibit the response of gastroscope insertion in the study of Ming Su et al. [23]. Another study by Meiyun Tan et al. [32] used the Dixon sequential method to determine the effective dose of esketamine for mitigating pain during propofol injection, and the initial dose of esketamine was 0.2 mg/kg. In our study, we select 0.3 mg/kg esketamine as the initial dose recommended by the instructions. This dose is commonly used in clinical practice and many studies [25, 26].

A reasonable choice of drug dosage will be necessary for decreasing the rate of adverse effects while maintaining the efficacy of the treatment. In order to determine the optimal amount of esketamine for preventing postoperative pain, four groups were divided by Yan-ling Ren and colleagues according to the different doses of esketamine(0 mg/kg, 0.2 mg/kg, 0.4 mg/kg, 0.6 mg/kg), and it reported that intravenous injection of esketamine 0.4 mg/kg before anesthesia induction was a suitable dose to reduce pain sensitivity in patients undergoing thyroidectomy without increasing adverse reactions [33]. As is well known, up-down sequential allocation is a simple, robust, and efficient method of identifying the median effective dose (ED50). In our study, the ED50 and ED95 of esketamine for preventing early postoperative pain were 0.301 mg/kg (95%CI: 0.265-0.342 mg/kg) and 0.379 mg/kg (95%CI: 0.340-0.618 mg/kg) respectively. Interestingly, the ED95 in this study is highly similar to the esketamine 0.4 mg/kg, suggesting our result has wide applicability and clinical practicability. However, the study by Meiyun Tan et al. [32] concluded that the ED50 and ED95 of esketamine for mitigating pain during propofol injection were 0.143 (0.120, 0.162) mg/kg and 0.176 (0.159, 0.320) mg/kg, respectively. This dose range is significantly different from our conclusion, and may be closely related to the type of pain. Meanwhile, another literature reported that the ED50 of esketamine was 0.143 mg/kg (95% CI 0.047–0.398 mg/kg) when combined with 3 mg/kg propofol for successful sedation in pediatric gastroscope insertion [23], which is significantly lower than the dose we measured. The possible reason is that the observation targets are children, and the assessment criteria are sedative effect rather than analgesia effect.

Postoperative pain after LC consists of three components: incisional pain (somatic), deep abdominal pain (visceral) and opioid-induced hyperalgesia (OIH). Incisional pain is a unique and common form of acute pain that may involve nociceptive, inflammatory, and neuropathic pain [3, 34]. The literature suggested that incisional pain could be reduced by incisional local anesthetics and dexamethasone [35]. In our study, after the 0.5% ropivacaine was applied to the incision for local infiltration anesthesia during skin sutures, there were no statistically significant differences between the two groups in incisional pain-related VAS score at resting and movement, and ropivacaine wore off after 8–12 h, suggesting it could effectively relieve incisional pain and last for 8–12 h.

Local infiltration block provides excellent analgesia for incisional pain, but unfortunately, visceral pain and opioid-induced hyperalgesia are still evident. Visceral pain is a complex, unpleasant feeling caused by trauma and inflammation that is generally described as dull, diffuse, and poorly localized, mainly transmitted by the autonomic nervous system, and is frequently accompanied by malaise and strong autonomic reflexes [36–38]. Visceral pain after LC primarily involves two aspects: cutting injury-induced pain and hyperalgesia. The cutting injury-related pain is thought to be mainly caused by the dissection of the gallbladder off the liver bed by electric knives. Visceral hyperalgesia and central sensitization also have been validated as the two most important visceral pain-related characteristics [9, 10, 38]. A study [39] in healthy volunteers showed that acutely increased cortisol enhanced pain sensitivity and impaired pain-related emotional learning within the visceral, but not the somatic pain modality. However, visceral pain is difficult to manage effectively, largely due to the complexities of visceral innervation, which leads to visceral pain-related pathophysiological factors being poorly understood. On the other hand, remifentanil, a u-opioid receptor agonist, is widely used for intraoperative analgesia because of its unique rapid metabolism and elimination without delaying postoperative recovery [14, 40]. It can provide adequate intraoperative analgesia, attenuating hemodynamic fluctuations from noxious stimuli during surgery. In our study, using remifentanil effectively contributed to containing the reflex increase in the mean blood pressure and heart rate induced by noxious stimuli such as skin incision or gallbladder dissection. However, exposure to high doses of remifentanil may reduce the pain threshold and induce hyperalgesia. For instance, C.-H. Koo and colleagues [13] reported that the pain threshold was significantly lower in the high-remifentanil group than in the low-remifentanil group, and naloxone reduced remifentanil-induced postoperative hyperalgesia. Based on the above analysis, opioid-induced hyperalgesia and visceral pain to some extent can be considered hyperalgesia and possess similar mechanisms [9, 10, 15, 38]. The underlying mechanisms of hyperalgesia are likely to be associated with upregulation or alteration of N-methyl-D-aspartate (NMDA) or pain-related receptors. Although the intraoperative use of low-dose naloxone reduced postoperative hyperalgesia [13], it was unsuitable for routine clinical practice because opioid receptor antagonists could exacerbate pain.

Ketamine, which works as an NMDA antagonist, has been shown to reduce incisional pain, visceral pain and hyperalgesia [4, 17, 41], as well as the postoperative consumption of morphine. Theoretically, as a dextral resolution of ketamine, the esketamine may have the same effect on early postoperative pain (incisional pain, visceral pain and hyperalgesia). Feng Liu and colleagues [42] reported that esketamine-based anesthesia (1 mg/kg) can alleviate postoperative pain and regulate the inflammatory reaction in children undergoing endoscopic plasma adenotonsillectomy. In our study, we found that there was a significant decrease in the total VAS score at resting during the awakening period after extubation in the esketamine group when compared with the control group (*p < 0.05*), suggesting esketamine could prevent early postoperative pain effectively, including visceral pain and hyperalgesia. However, there were no statistically significant differences between the two groups for the total VAS score during cough and the numbers requiring rescue analgesia during the awakening period (*p > 0.05*), and there are three possible explanations for these: (1) the esketamine dose too low in some patients of the Group E; (2) the sample size is too small and insufficient number of events; (3) the cough reflex exacerbate the incisional pain and visceral pain.

Esketamine is a dissociative anesthetic with sympathomimetic and broncho-dilating properties, similar to ketamine. Recent reports found that 14/70 patients experienced treatment-emergent transient hypertension after intravenou*s* esketamine, and it was significantly higher than the baseline value [43], which is mainly related to the sympathetic nerve-induced release of catecholamine [44]. In our study, there was a decrease in heart rate and mean arterial pressure in esketamine group at T1 compared to the basal values and control group, which did not increase due to using esketamine. The reasons for this may be multiple: (1) other anesthetics have vasodilatory effects; (2) lack of circulating blood volume due to fasting; (3) there was a more thorough blocking for the nociceptive stimuli after using esketamine. Therefore, the changes in vital signs during anesthesia need further study, when esketamine is combined with other anesthetics. On the other hand, using esketamine did not delay waking up after anesthesia and also did not increase the frequency of adverse reactions.

Our study has some limitations. Firstly, the sample size was set according to the up-down allocation methodology rather than the sample size estimation. Secondly, the comparison of VAS scoring between the two groups had some limitations because the esketamine group used different doses. Finally, this study only examined early postoperative pain to ensure adequate analgesia during the operation, and did not evaluate long-time postoperative analgesia of esketamine.

## Conclusions

In conclusion, our study showed that the ED50 and ED95 of esketamine for preventing early postoperative pain were 0.301 mg/kg (95%CI 0.265 ~ 0.342 mg/kg) and 0.379 mg/kg (95%CI 0.340 ~ 0.618 mg/kg) respectively. Intraoperative esketamine effectively dampened intraoperative noxious stimuli and had no impact on awakening from anesthesia, with a favorable safety profile.

## Data Availability

The datasets used and/or analyzed during the current study are available from the corresponding author on reasonable request.

## Abbreviations

ASA: American Society of Anesthesiologists
BIS: The bispectral index
BMI: Body mass index
EC95: 95% effective dose
ED50: Median effective dose
HR: Heart rate
LC: Laparoscopic cholecystectomy
MAP: Mean arterial pressure
OIH: Opioid-induced hyperalgesia
PACU: Postanesthetic care unit
PONV: Postoperative nausea and vomiting
VAS: Visual analogue scale/score
